# Effects of Intradermal Radiofrequency Treatment and Intense Pulsed Light Therapy in an Acne-induced Rabbit Ear Model

**DOI:** 10.1038/s41598-019-41322-x

**Published:** 2019-03-25

**Authors:** Joon Seok, Jong Hwan Kim, Jae Min Kim, Tae Rin Kwon, Sun Young Choi, Kapsok Li, Beom Joon Kim

**Affiliations:** 1 Department of Dermatology, Chung-Ang University College of Medicine, Seoul, Korea; 2 Graduate School of Medical Science and Engineering, KAIST, Daejeon, Korea; 3 Department of Dermatology, Seoul Paik Hospital, Inje University, Seoul, Korea

## Abstract

Acne vulgaris is a common condition that can have psychologically deleterious effects. Since current treatments carry the risks of antibiotic resistance or teratogenicity, novel treatment modalities are under investigation. Our study investigated the efficacy of intradermal radiofrequency treatment (RF) and intense pulsed light (IPL) in the treatment of acne vulgaris in a rabbit ear model. We evaluated the effectiveness of IPL, RF, and a combination treatment on cultured *Cuticobacterium acnes* strains in an induced rabbit ear model, according to clinical outcomes as well as histological and immunological approaches. We found that RF treatment markedly decreases papule volume, while IPL appears to have an immunomodulatory effect. In combination, the two have an additive effect in treatment. These findings suggest that combination of RF and IPL may be an effective therapeutic option for the treatment of acne vulgaris.

## Introduction

Acne vulgaris is a common inflammatory skin condition characterized by the development of comedones, papules, and pustules. In serious cases, nodules and deep pustules as well as cysts may also develop. Acne vulgaris can lead to substantial social and psychological problems including reduced self-esteem, heightened anxiety, depression, and mood disorders^1–3^. Its chief clinical treatments include antibiotics and isotretinoin, but these can be limited in their usefulness and acceptability^4,5^. Furthermore, treatments are often difficult and inconvenient to use, time-consuming, and can be expensive, and post-treatment relapse is common; as a result, compliance may be poor^6^. In isotretinoin treatment, relapse rates are relatively low if the drug is administered with a sufficient cumulative dose of over 120–150 mg/kg^7^, but some patients are unable to tolerate side effects such as cheilitis, xerosis, epistaxis, elevated liver enzymes, and elevated blood cholesterol. The drug is also contraindicated in many patients due to its teratogenic effects^8^. In antibiotic treatment, the development of antibiotic-resistant bacteria is an ongoing concern^9^. As such, novel treatment modalities are needed in order to provide alternatives and complements for the existing therapeutic options.

Sebaceous glands and *Cuticobacterium acnes* bacteria are central to the development of acne. Proliferation of *C. acnes* may contribute to inflammation by activating toll-like receptors (TLR)-2 and TLR-4, which in turn leads to the release of pro-inflammatory cytokines including interleukin (IL)-1α, IL-1β, IL-6, IL-8, IL-12, and tumor necrosis factor-alpha (TNF-α), as well as transforming growth factor-beta (TGF-β)^10–12^. TGF-β appears to be an essential anti-inflammatory and immunomodulatory cytokine, which can inhibit CD4+ T-lymphocyte-mediated inflammation occurring in the early phase of acne lesion development^13,14^. It can also contribute to keratinocyte growth arrest and therefore disturb microcomedone formation^15^, and plays a key role in wound recovery^16–18^. *C. acnes* increases functional activity in the sebaceous glands and promotes sebum production, and is thought to be involved in acne by promoting the injury of tissue due to the release of enzymes such as lipases^19^. These catalyze the dissolution of sebum triglycerides to glycerol and free fatty acids, leading to the influx of polymorphonuclear neutrophils via chemotaxis^12^. As such, preventing inflammation could be an effective treatment for acne by inhibiting *C. acnes* colonization^20,21^.

Peroxisome proliferator-activated receptor-gamma (PPAR-γ) is a lipid-activated nuclear hormone receptor participating in sebaceous lipogenesis and differentiation in addition to inflammation^22–24^. Sebaceous glands express PPAR-γ, and a number of eicosanoids are capable of triggering this transcriptional factor *in vitro*^22,24^. In both acne lesions and non-acne lesions of acne patients, PPAR-γ is either low or absent in the sebaceous glands^25^. We suggest that when PPAR-γ is absent, sebaceous glands’ inflammatory regulation fails, and auto-inflammatory reactions are switched on. Furthermore, lipid production within acne sebaceous glands is greater than in healthy sebaceous glands. This suggests that in sebaceous glands of acne lesions, the primary effect of PPAR-γ may be to regulate inflammation, and possibly to maintain balanced lipid production^25^.

Because of the drawbacks associated with antibiotics and isotretinoin use, many acne patients seek minimally invasive treatments such as intense pulsed light (IPL) and radiofrequency (RF) treatment to alleviate inflammatory acne and its sequelae. IPL is commonly used to treat pigmentary disorders and for epilation. Although it is sometimes used in photodynamic therapy of acne, its performance is questionable, and its efficacy and applicability require further investigation^26–28^. Similar questions apply to intradermal RF treatment^29,30^. Therefore, we aimed to assess the effectiveness of IPL, RF, and a combination of the two on *C. acnes* strains and using an acne-induced rabbit ear model.

## Results

### Scanning electron microscopy (SEM)

**Figure 1:**
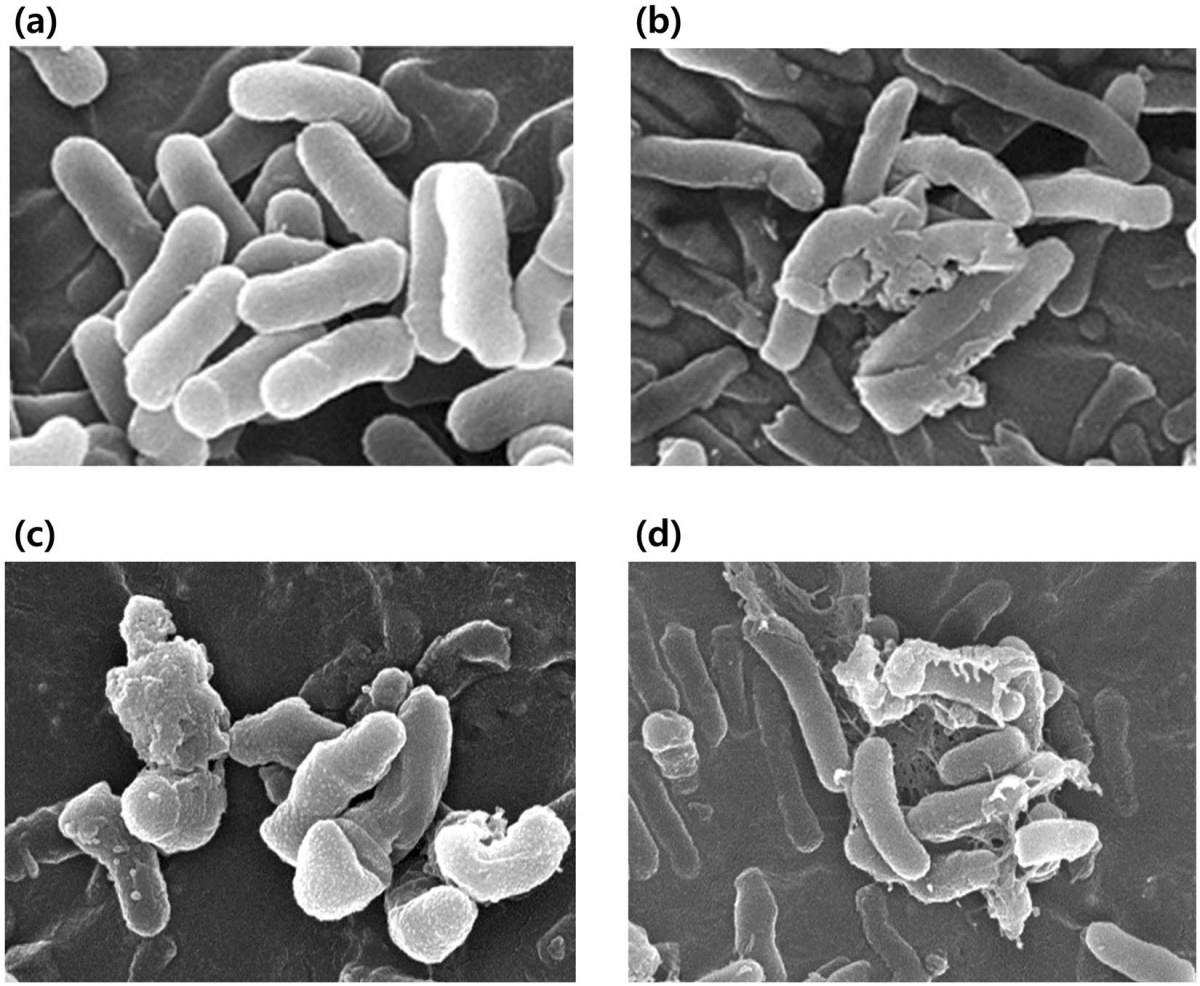
Scanning electron microscopy (SEM) images. (**a**) Untreated control. Bacteria appeared as expected, with a smooth and rounded surface. (**b**) Radiofrequency (RF) treated group (1 MHz, 36 W, 400 ms, 1 shot). (**c**) Intense pulsed light (IPL) treated group (450–1000 nm, 10.8 J/cm^2^, three pulses). RF- and IPL-treated bacteria had a withered and recessed surface. (**d**) Combination treatment of both RF and IPL. *C. acnes* exhibited greater damage under combination treatment in comparison to either RF or IPL treatment alone.

SEM was used to examine *C. acnes* that had been untreated or treated with RF, IPL, or RF + IPL. Untreated bacteria appeared as expected, with a smooth and rounded surface (Fig. 1a). Treated bacteria had a more withered and recessed surface (Fig. 1b,c). Differences between bacteria treated by single therapies, such as RF or IPL, and those treated by combination therapy, were apparent. *C. acnes* were much damaged by the combination treatment in comparison to either RF or IPL treatment (Fig. 1b–d).

### Morphological changes and histological analysis

**Figure 2:**
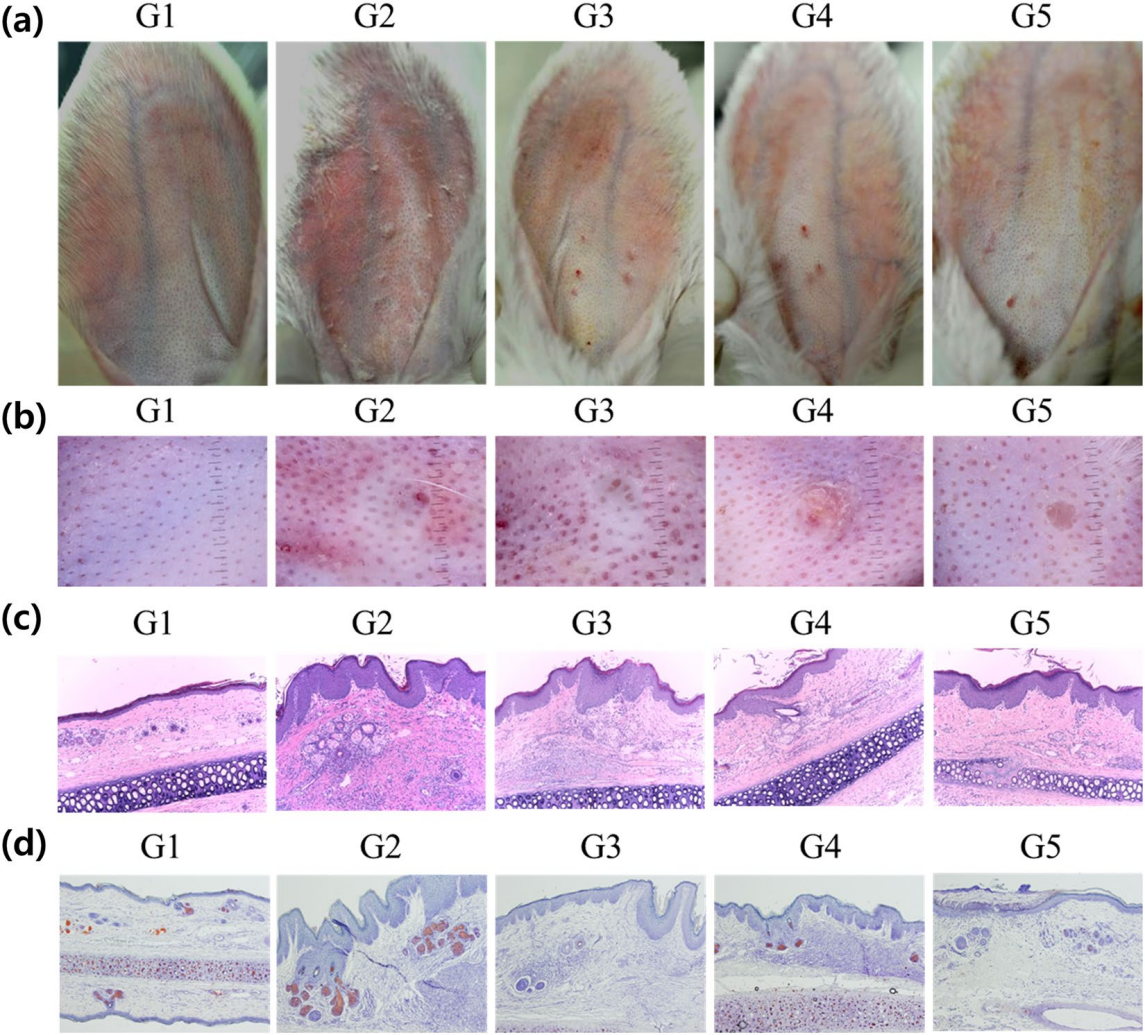
Morphology and histology after seven days. (**a**) Digital camera images. (**b**) Phototrichogram evaluation. (**c**) H&E (×100). (**d**) Oil red O stain (×100). Seven days after treatment, the treatment groups displayed greater improvement than the positive control group, without adverse effects. There were marked infiltration of inflammatory cells around sebaceous glands in the G2 positive control. In G3 and G5, destruction of the sebaceous glands was evident. Combination treatment produced less inflammatory cell infiltration compared to treatment with RF or IPL alone. The epidermis was intact in all treatments. In Oil red O staining, G3 and G5 had no observable sebaceous glands. The size of sebaceous glands was significantly reduced in G4.

The epidermis remained intact in all treatments (Fig. 2c). Seven days after treatment, all treatment groups displayed improvement without adverse effects in comparison to the positive control group (Fig. 2a). Total lesion count indicated that G3, G4, G5 had markedly improved, with reduction rates of 59.6% for G3, 24.35% for G4, and 67.80 for G5 (Table 1). Phototrichogram evaluation indicated an identical outcome (Fig. 2b). H&E stained sections prepared from samples obtained seven days after treatment showed marked infiltration of inflammatory cells around the sebaceous glands in the G2 positive control. In G3 and G5, destruction of the sebaceous glands was evident. In the G5 combination treatment, there were fewer and less pronounced inflammatory cell infiltrations compared RF or IPL alone. There were no Oil Red O-stained sebaceous glands in the G3 or G5 groups. Sebaceous gland size in G4 was significantly reduced compared to that in G2 (Fig. 2d).

**Table 1:** Lesion number count in an acne-induced rabbit ear model.

	Baseline	After treatment	Reduction rate (%)	P-value
G1	0.0 ± 0.0	0.0 ± 0.0	0	
G2	17.00 ± 1.00	19.0 ± 1.00	−11.76	0.0705
G3	19.00 ± 1.00	7.7 ± 1.53	59.65	0.0004^***^
G4	17.67 ± 0.58	13.33 ± 0.58	24.35	0.0008^***^
G5	19.67 ± 0.58	6.33 ± 2.08	67.80	0.0009^***^

Total lesion count showed that G3, G4, and G5 improved significantly. *p < 0.05, ***p < 0.0005.

**Figure 3:**
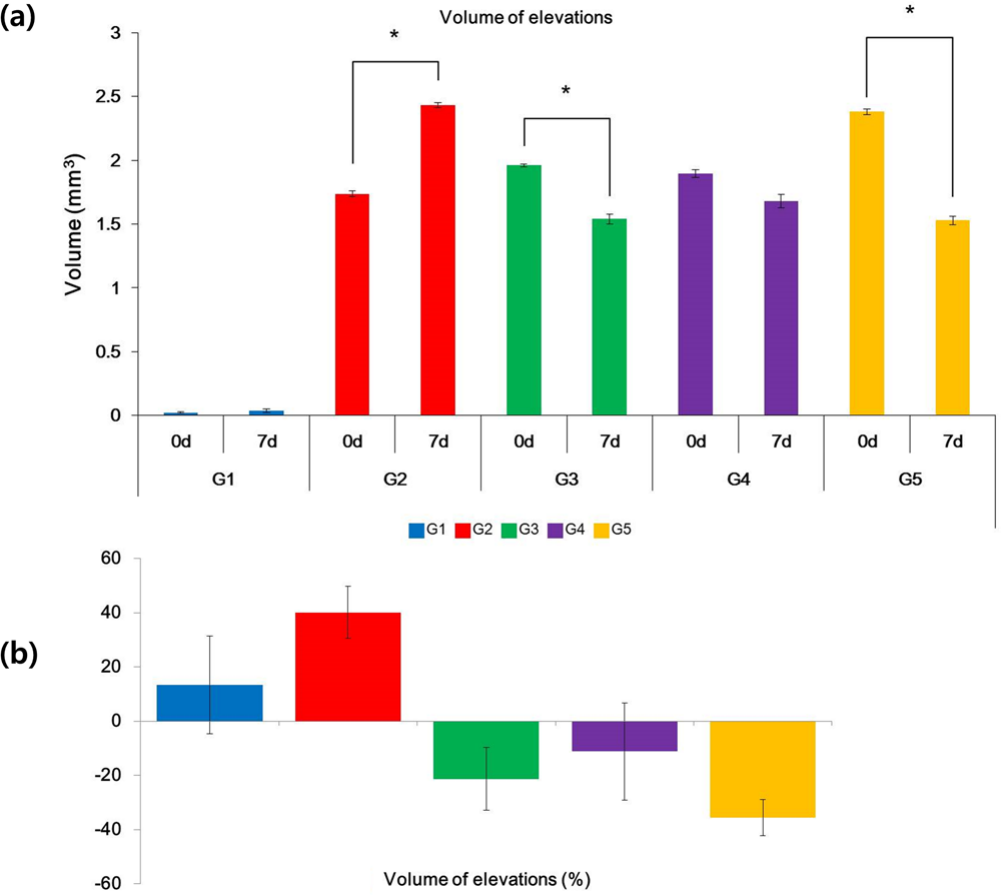
Objective three-dimensional (3D) volume change measurement. Using the 3D skin measurement system, volumes can be measured on areas prior to and after treatment. (**a**) Volumetric analysis (mm^3^). There was increased volume in the untreated group, G2, while significant volume reduction was apparent in G3 and G5. G4 displayed reduced volume but the reduction was not statistically significant. (**b**) Volumetric analysis (%). When the volume change was compared between groups, G3 and G5 decreased in volume by 21.30% and 35.61%, respectively. The volume of G4 groups decreased by 11.18% (*p < 0.05).

According to the objective three-dimensional scanning measurements, there was increased volume of acne papules in the untreated group, G2 (p = 0.0461), while a significant reduction in volume was apparent in G3 (p = 0.0382) and G5 (p = 0.0326). G4 displayed reduced volume, but this was not statistically significant (p = 0.7792; Fig. 3a). When the volume change was compared between groups, G3 and G5 were found to have decreased significantly by 21.30% (p = 0.0137) and 35.61% (p = 0.0170), respectively. The volume in the G4 group likewise decreased significantly by 11.18% (p = 0.0672; Fig. 3b).

### Immunohistochemistry and immunofluorescence

**Figure 4:**
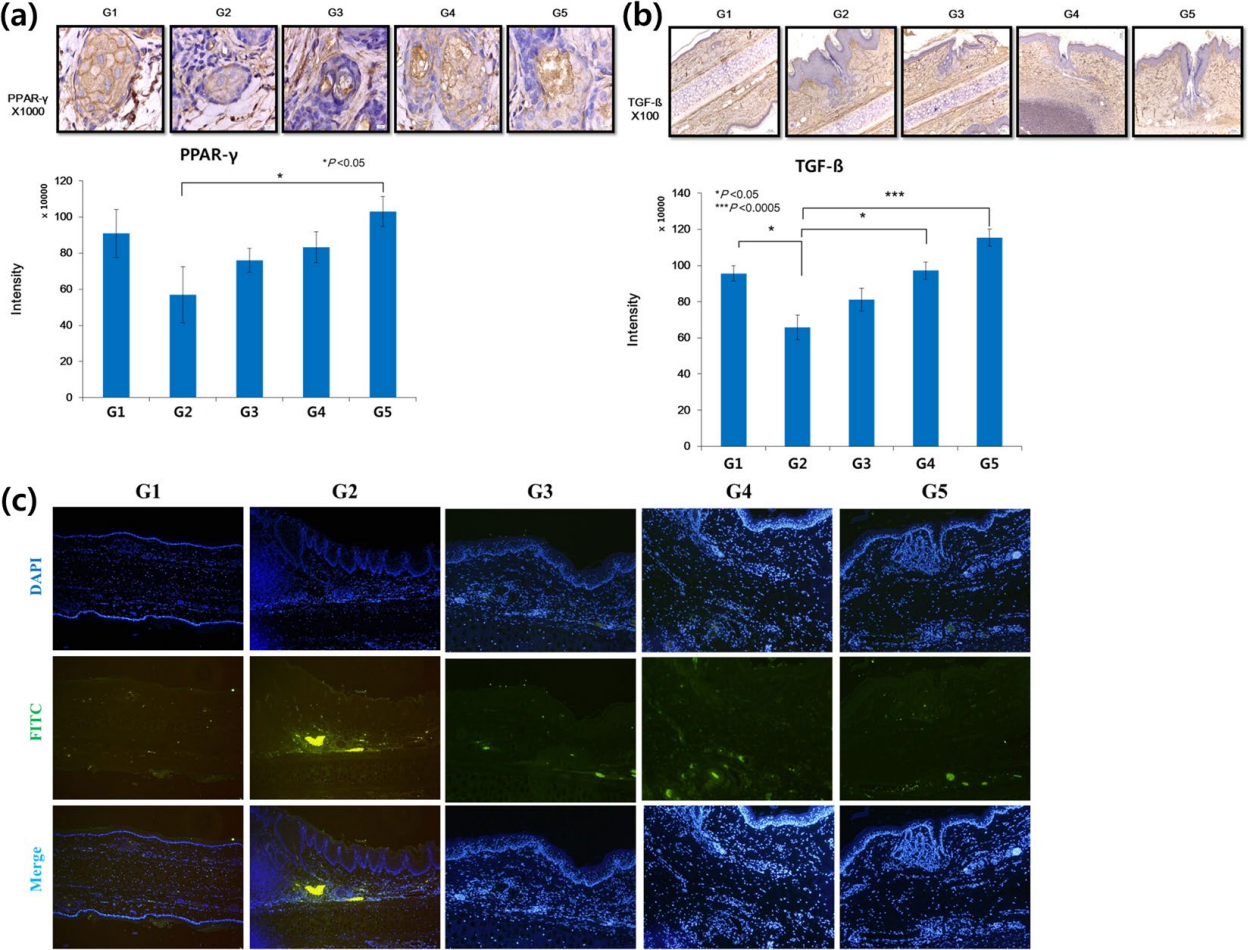
Immunohistochemistry and immunofluorescence. (**a**) PPAR-γ staining indicated a greater, but not statistically significant, decrease in expression in G2 compared to G1. PPAR-γ expression was increased in G3 compared to G2, and expression was increased slightly further in G4. However, there were no significant differences between the degree of expression between G2 and G3 or G4. G5 was significantly different from G2 (PPAR-γ, ×1000). (**b**) TGF-β expression was significantly lower in G2 compared to G1. TGF-β expression in G3 increased in comparison to G2, but not significantly so. TGF-β expression in G4 and G5 was significantly different from G2, with an especially large difference in the case of G5 (TGF-β, ×100) (**c**) Immunofluorescence staining for TNF-α (green) was performed. Nuclei were counterstained with DAPI (blue) (×200). Immunofluorescence indicated a prominent deposition of TNF-α in the area surrounding the sebaceous gland in G2. Immunostaining revealed that the treatment groups (G3-G5) had notably reduced TNF-α expression levels compared to the positive control group (G2). *p < 0.05, ***p < 0.0005.

PPAR-γ staining indicated a greater, but not statistically significant, decrease of expression in G2 compared to G1 (p = 0.1359). PPAR-γ expression showed a greater increase in G3 compared to G2, and greater still in G4. However, there were no significant differences in expression levels between G2 and G3 or G4 (G2-G3: p = 0.2936, G2-G4: p = 0.1759). G5 was found to be significantly different compared to G2 (p = 0.0306; Fig. 4a).

TGF-β staining indicated that its expression was significantly decreased in G2 compared to G1 (p = 0.0057). In G3, TGF-β expression increased in comparison to G2, but not significantly (p = 0.1341). TGF-β expression in G4 and G5 was significantly different compared to G2, with an especially large difference in the case of G5 (G2-G4: p = 0.0050, G2-G5: p = 0.0003) (Fig. 4b).

Immunofluorescence indicated a prominent deposition of TNF-α in the area surrounding the sebaceous gland in G2. Immunostaining showed that the treatment groups G3-G5 had notably reduced TNF-α expression levels compared to the G2 positive control group (Fig. 4c).

## Discussion

Light and laser therapies for treating acne are effective with infrequent adverse effects^5,27,29^. Nevertheless, the exact processes underlying their actions remain unclear. Previous studies have shown that RF reduces sebum production through the selective electrothermolytic destruction of sebaceous glands^29–31^, wherein the glands are subsequently replaced by fibrosis, leading to dermal contraction and resulting in skin tightening and pore size reduction^31^. Our results demonstrate that RF energy can effectively destroy the sebaceous gland. However, histological examination could not confirm that RF induced fibrosis. At seven days post-treatment, only the infiltration of inflammatory cells would be observed. However, as there was an increase in the expression of TGF-β, it is likely that fibrosis and dermal remodeling will occur over time^32^. SEM findings indicate that the energy released during RF treatment can successfully destroy *C. acnes*. Objective three-dimensional measurements allowed us to confirm that the volume of acne lesions was significantly lower after RF treatment. This likely occurred as a result of sebaceous gland destruction and the expulsion of debris from the skin.

Seven days after IPL treatment, the decrease in acne lesion volume was lower compared to the volume decrease achieved using RF. This may be because of a photodynamic effect, wherein IPL activates endogenous porphyrins created by *C. acnes* to induce TGF-β^13^, rather than cause destruction of the sebaceous gland as in RF. Additionally, the IPL spectrum includes red light, which mediates the reduction of inflammation and promotion of wound healing and can be beneficial for acne^33–35^. SEM confirmed that the surface of *C. acnes* was destroyed by IPL in this experiment. These beneficial effects of IPL may bring about clinical improvements, but these do not seem to be sufficient to reduce the number and size of lesions to the same degree as RF treatment over 7 days.

Reduced PPAR-γ expression levels in the acne-induced model compared to the normal control were observed. Compared to the positive control, RF and IPL treatment resulted in increased PPAR-γ expression level, though not significantly so. When the two treatments were provided in combination, there was a statistically significant outcome. Therefore, it appears that the increase of PPAR-γ regulates inflammation in acne lesions and aids rapid treatment outcome and recovery, particularly under combination treatment.

Lasers, including IPL, may have an immunomodulatory effect in the treatment of inflammatory acne. A previous study showed transcription and expression of TGF-β for a week following RF treatment, with neocollagenesis via increased expression of type I and III collagen and upregulation of type I collagen mRNA apparent within the affected tissue^36^. In our study, the TGF-β expression level in the acne-induced model was significantly decreased compared to the normal control expression levels. In the case of IPL treatment, TGF-β expression was significantly increased, with even greater level of expression achieved using the combined RF and IPL treatment. This suggests that the combination treatment could be useful in acne treatment via an immunomodulatory effect elevating TGF-β expression.

Previous studies have suggested that downregulation of TNF-α, rather than IL-8 or TLR-2, is the most likely therapeutic target for IPL^37,38^. Use of the RF on acne also resulted in attenuated TNF-α release in an acne model^29^. We performed immunofluorescence microscopy to assess the release of TNF-α in the control and treatment groups. RF, IPL, and a combination treatment of these therapies modified TNF-α expression within acne lesions. We believe that treating lesions with RF or IPL can reduce TNF-α, and will contribute to mitigating the progression of acne lesions.

The activation of NLRP3 inflammasomes in monocytes in the skin is a major source of pathogenesis of acne vulgaris^39–41^. Local inflammasomes in sebaceous units induced by *C. acne* mediate the production of the major inflammatory cytokine IL-1β, which promotes severe localized inflammation in pilosebaceous glands. Since TNF-α is suggested as an upstream mediator of IL-1β, reduced TNF-α expression with combination treatment of RF and IPL may reduce the secretion of IL-1β. Therefore, decreasing the effects of NLRP3-inflammasome may decrease inflammation induced by *C. acnes*.

There are some limitations to our study stemming from our use of a rabbit ear assay (REA), which is commonly used to ascertain compound comedogenicity^42^. Rabbit inner ear follicles are similar to human follicles in that both possess small pili and large adjacent sebaceous glands, and the REA is sensitive to the degree that only two weeks of applying a test agent can produce erythema, desquamation, and follicular keratosis, making the model useful in studies of the causes of acne^43,44^. During the preliminary animal study performed to establish the assay, we applied oleic acid for 6 weeks and injected *C. acne*. When we ceased application after the development of acne lesions, the size of the papules continued to increase for one week, as can be seen in human patients. Thereafter, spontaneous regression was evident (data not shown). However, this model is not entirely homologous concerning the characteristics of their human equivalents, with intrinsic differences in skin permeation and metabolism^45,46^. Rabbit ear hyperkeratosis is different from the comedo of acne vulgaris in several ways. First, the REA features little bacterial colonization. Second, inflammatory lesions do not emerge since follicular wall rupture does not occur. Third, the horn is not as densely packed. Fourth, mechanical expulsion is easier in the case of the rabbit^47^. Because acne lesions cannot be maintained longer than a week in the rabbit model, the influence of *C. acnes* is reduced with time, as continued bacterial colonization is unsuccessful. This may be due to a reduction of inflammation because of the absence of rupture of the follicular wall. Therefore, the REA is limited in that it can only be used to observe the effectiveness of treatments in the short term, as in our study’s one-week timeframe. It is also possible that our observed outcomes may have been better than potential clinical results from a human patient’s acne lesions, since debris can escape more easily from the rabbit model than from human skin during microneedle puncture during RF treatment.

In conclusion, RF appears to notably reduce lesion volume when used on acne, while IPL appears to have an immunomodulatory effect. We also demonstrated that the two treatments in combination produce an additive effect. In the case of RF, although it did not produce a statistically significant outcome, there appears to be an additional slight immunomodulatory effect. When performing RF treatment, one can expect that the wound caused by needle insertion for treatment would heal quickly due to the TGF-β enhancement effect of the IPL. In addition, although IPL treatment is less destructive of the sebaceous gland than RF, it is believed to aid the treatment of acne through increasing TGF-β, decreasing TNF-α, and increasing PPAR-γ. Although IPL did not yield a large reduction in volume of acne lesions themselves in seven days, its immunomodulatory effect is likely to aid the overall treatment of acne. Future trials should investigate whether it is best to perform IPL and RF at the same time, or whether they should be performed in any staggered intervals.

## Methods

### Ethics statement

Forty 4-month-old male New Zealand white rabbits (2 to 2.5 kg, Saeronbio, Uiwang, Korea) were used for animal experiments. They were housed under standard temperature and humidity in the specific pathogen-free facilities of Chung Ang University. All animal procedures were performed in accordance with the Guidelines for Care and Use of Laboratory Animals of Chung-Ang University and approved by the Animal Ethics Committee of Chung-Ang University IACUC (Approval No. 2016-00090).

### *C. acnes* culture

*C. acnes* (ATCC 11827) was the control strain for anaerobe identification. *C. acnes* cultures were grown on modified reinforced clostridial agar/broth medium in an anaerobic atmosphere of 80% N_2_, 10% CO_2_, and 10% H_2_ at 37 °C for 48 to 72 h. *C. acnes* at 10^7^ colony-forming units (CFU) per 20 µL were heat inactivated at 95 °C for 5 min, then suspended in an appropriate amount of phosphate buffered saline (PBS) for experiments.

### Scanning electron microscopy (SEM) of treated bacterial cultures

*C. acnes* preparations as described above were sorted into four treatment groups, with one receiving no treatment, one receiving RF with one pulse of 1 MHz at 36 W for 400 ms, one receiving IPL at 450–1000 nm at 10.8 J/cm^2^ with three pulses, and one group receiving both IPL and RF. Both IPL and RF were delivered using a DermAkne instrument (Huons, Seongnam, Korea). This device has dual mechanism composed of a monopolar intradermal RF and IPL. Treated *C. acnes* (2 × 10^7^/mL) were fixed with 2% glutaraldehyde in PBS at room temperature for 30 minutes, washed, suspended in PBS, and retained on a microporous filter. Each filter coated with *C. acnes* was dehydrated in a graded ethanol series (50%, 75%, 95%, and 100%, each for 15 minutes) followed by hexamethyldisilazane (50%, 75%, and 95%, each for 30 minutes). Samples were then incubated overnight in 100% hexamethyldisilazane. Each sample was coated with platinum and observed using a JSM-PH SEM microscope (Jeol, Tokyo, Japan).

### Acne-induced rabbit ear model

Using a 30-gauge needle, a suspension of *C. acnes* (10^7^ CFU in 20 µL PBS) was injected at six sites in the external ear of the experimental rabbits, avoiding contact with blood vessels. These were followed by application of 50% oleic acid in Macrogol 400 (polyethylene glycol, MW 400; Sigma-Aldrich, St Louis, MI, USA) performed once a day for 42 days.

### Treatment with monopolar RF & IPL

Five treatment groups were evaluated in rabbits: a normal control group (G1), an acne-induced positive group (G2), a monopolar RF treated group (G3), an IPL-treated group (G4), and a group treated with IPL + RF (G5) (Table 2), with eight rabbits per group. Rabbit ears were investigated before and after monopolar RF and IPL treatment. The animals were sacrificed and analyzed seven days after treatment. In the case of RF, the sebaceous gland was selectively destroyed without damage to the epidermis by heat transfer using a 1.2 mm micro-insulated needle (MN), which penetrated the skin and delivered energy to the rabbit ear at 1 MHz, 36 W for 400 ms. IPL was applied in three pulses at 450–1000 nm at 10.8 J/cm^2^.

**Table 2:** Treatment groups in an acne-induced rabbit ear model.

	*Cuticobacterium acnes* injection + oleic acid topical treatment	Treatment
G1	(—)	None
G2	(+)	None
G3	(+)	Monopolar RF device^†^
G4	(+)	IPL^‡^
G5	(+)	Monopolar RF device + IPL^#^

Five treatment groups were evaluated, including a normal control group (G1), an acne-induced positive control group (G2), a monopolar RF-treated group (G3), an IPL-treated group (G4), and an IPL + RF-treated group (G5). Eight rabbits were treated in each group. Rabbit ears were investigated before and after treatments. ^†^RF: 1 MHz, 36 W, 1.2 mm needle, ^‡^IPL: triple pulse, 450−1000 nm, 10.8 J/cm^2^, ^#^RF + IPL: same protocol as Groups 3 and 4.

### Clinical evaluation

Images were taken daily with a 3000D digital camera (Canon Inc., Tokyo, Japan). Changes in inflammatory lesion (papules, pustules, and nodules) and total lesion counts (inflammatory lesions and noninflammatory lesions) were noted for seven days post-treatment. Lesion counts were recorded for the whole ear canal area in both ears of each rabbit. The total number of lesions was considered to be 100% and any decrease in the number of lesions was calculated accordingly and regarded as percent improvement. A phototrichogram system (Folliscope, Lead-M, Seoul, Korea) was used to examine changes to the skin after treatment. To evaluate the progression and efficacy of the treatments, volume covered by follicular orifices was calculated using digital image analysis performed using Antera 3D software (Miravex Limited, Dublin, Ireland)^48,49^.

### Histologic and immunologic measurements

Specimens of inflammatory acne lesions (papules) in the rabbit ear and healthy skin samples of the adjacent area were obtained using 8 mm biopsy punches seven days after treatment. Each sample was immediately placed in 10% neutral buffered formalin dehydrated with a graded ethanol series (70% for 2 h, 80% overnight, 90% for 2 h, and 100% for 2 h) and embedded in dimethylbenzene-paraffin. Sections (5 µm) were cut, placed onto slides, and dried in a 70 °C chamber for 40 min. After being dewaxed with ethanol, the sections were soaked with hematoxylin, water and 1% hydrochloric ethanol at room temperature for 5 min, 5 min, and 3 sec respectively, and rinsed with water for 20 min. The slices were subsequently stained with eosin for 3 min and dehydrated with a graded series of ethanol (75%, 85%, and 95%, each for 2 min, and 100% twice for 5 min). Additionally, skin biopsy samples were stored at ‒80 °C and placed in a cryomold at optimum cutting temperature (OCT) (Tissue Tek, SakuraFinetekInc., Torrance, CA, USA). Subsequently, 10-µm-thick sections were stained with Oil red O. The stained tissues were observed under a DP73 microscope (Olympus, Tokyo, Japan).

For immunohistochemistry, slides were blocked in an UltraVision Protein block (Thermo Scientific, Waltham, MA) for 1 h and then incubated with rabbit monoclonal antibodies against PPAR-γ (ab45036, 1: 200; Abcam, Cambridge, UK) and rabbit monoclonal antibodies against TGF-ß (ab190503, 1: 200; Abcam) at 4 °C overnight. The slides were washed and incubated with secondary horseradish peroxidase (HRP)-conjugated antibody using the UltraVision Quanto Detection System HRP Polymer Detection Kit (Thermo Scientific, Waltham, MA). After washing with Tris-buffered saline containing Tween-20, slides were stained using a 3,3′-diaminobenzidine-based Quanto kit (Thermo Scientific, Waltham, MA). Slides were then counterstained with hematoxylin. Slides stained with TGF-ß and PPAR-γ were selected from raw photographs and saved as jpeg images using image processing software (Adobe Photoshop, San Jose, CA). A subset of colors representing the brown-stained area was selected from raw photos and saved as jpeg images. Dye intensity was measured for each image using another image analysis program (Image Pro, Media Cybernetics, Rockville, MD), and the stained area was calculated and expressed in pixels.

For immunofluorescence, slides were blocked in PBS containing 0.2% Triton X-100 and normal horse serum at room temperature for 2 h and then incubated with mouse monoclonal antibodies against TNF-α (sc-12744, Santa Cruz Biotechnology, Santa Cruz, CA) at 1:100 dilution and incubated at 4 °C overnight. The slides were washed and incubated with fluorescein isothiocyanate-conjugated anti-Armenian hamster secondary antibodies (sc-2446, 1:200; Santa Cruz Biotechnology, Santa Cruz, CA) at room temperature for 30 min. Slides were then counterstained with 4′,6-diamidino-2-phenylindole (DAPI) for 5 min.

### Statistical analysis

The mean percentage improvement was calculated for each group of subjects and used for statistical analysis. At the end of the study, the data were analyzed using SPSS 19 (SPSS, Chicago IL). Comparisons between groups were analyzed using a one-tailed Student’s t-test. All data are presented as a mean with standard deviation (SD). Differences were regarded as statistically significant at p < 0.05.
